# The Influence of Prescribed Fire, Habitat, and Weather on *Amblyomma* *americanum* (Ixodida: Ixodidae) in West-Central Illinois, USA

**DOI:** 10.3390/insects9020036

**Published:** 2018-03-22

**Authors:** Mary E. Gilliam, Will T. Rechkemmer, Kenneth W. McCravy, Seán E. Jenkins

**Affiliations:** 1 Department of Biological Sciences, Western Illinois University, 1 University Circle, Macomb, IL 61455, USA; beths77@illinois.edu (M.E.G.); WT-Rechkemmer@wiu.edu (W.T.R.); SE-Jenkins@wiu.edu (S.E.J.); 2 Illinois Natural History Survey, Prairie Research Institute, 1816 South Oak Street, Champaign, IL 61820, USA

**Keywords:** litter cover, lone star tick, microclimate, oak woodland, prescribed burning, tick ecology, vegetation structure

## Abstract

The distribution of *Amblyomma americanum* (L.) is changing and reports of tick-borne disease transmitted by *A. americanum* are increasing in the USA. We used flagging to collect ticks, surveyed vegetation and collected weather data in 2015 and 2016. *A. americanum* dominated collections in both years (97%). Ticks did not differ among burn treatments; however, tick abundance differed between years among total, adult, and larval ticks. Habitat variables showed a weak negative correlation to total ticks in respect to: Shannon diversity index, percent bare ground, perennial cover, and coarse woody debris. Nymphal ticks showed a weak negative correlation to percent bare ground and fewer adults were collected in areas with more leaf litter and coarse woody debris. Conversely, we found larvae more often in areas with more total cover, biennials, vines, shrubs, and leaf litter, suggesting habitat is important for this life stage. We compared weather variables to tick presence and found, in 2015, temperature, precipitation, humidity, and sample period influenced tick collection and were life stage specific. In 2016, temperature, precipitation, humidity, wind, and sample period influenced tick collection and were also life stage specific. These results indicate that spring burns in an oak woodland do not reduce ticks; other variables such as habitat and weather are more influential on tick abundance or presence at different life stages.

## 1. Introduction

The blacklegged tick (*Ixodes scapularis* Say), lone star tick (*Amblyomma americanum* L.) and American dog tick (*Dermacentor variabilis* Say) are known to vector a variety of pathogens that cause illnesses in humans and animals [1,2,3,4]. Reports of tick-borne disease have increased worldwide, and new diseases continue to emerge [3,4,5]. Lyme disease is the most common tick- borne illness in the USA [6]. Although *A. americanum* are not competent vectors of Lyme disease (*Borrelia burgdorferi*), the bacterium causing this disease has been previously found in these ticks [7,8,9]. In the USA, *A. americanum* is known to vector several pathogens including human monocytotropic ehrlichiosis (*Ehrlichia chaffeensis*), canine and human granulocytic ehrlichiosis (*Ehrlichia ewingii*), tularemia (*Francisella tularensis*), southern tick-associated rash illness (STARI) (*Borrelia lonstari*), and a new *Phlebovirus*, Heartland virus [10,11,12]. Of these diseases, canine and human granulocytic ehlichiosis, monocytotropic ehrlichiosis and tularemia have been reported in Illinois [13].

*A. americanum* are known to be aggressive, generalist feeders [14] and historically have been found in higher densities in the southern and eastern USA [15,16]. The geographic range of the lone star tick has recently expanded and populations of *A. americanum* are increasing in the midwestern USA, especially in Illinois [15,17]. Populations of *A. americanum* are influenced by synergistic ecological factors including climate, habitat, and host dynamics [15,18,19].

*A. americanum* feed on a variety of mammals that inhabit woodlands, which consequently affects their distribution [20,21]. Adults are most commonly found feeding on large mammals such as white-tailed deer (*Odocoileus virginianus* Zimmerman) [22,23]. Host behavior is influenced by a variety of abiotic and biotic factors, including vegetation which varies seasonally and annually and causes hosts to be drawn to some plant communities more often than others [24]. For example, *O. virginianus* prefer habitats with greater vegetation density, due to the cover it provides [20]. In an agriculturally dominated, fragmented landscape (such as Illinois), harvested row crops and mast production in fall affect deer movement and as a result affect the distribution of ticks [25,26].

Vegetation structure can also influence the temperature, humidity, and air flow near the woodland floor [27,28,29]. These variables are often examined in tick population studies because they have been shown to affect tick survival and questing behavior [30,31]. Ticks have a high surface-to-volume ratio and can desiccate quickly when temperatures are high and humidity is reduced [32], although sensitivity to changes in environmental conditions varies among tick species and may be life stage-specific [33,34]. Larval ticks are at the greatest risk of desiccation and must descend into the litter layer frequently during unfavorable microclimate conditions [34]. Over time, layers of leaves, twigs and other detritus accumulate to provide a moist favorable substrate for ticks not actively questing for hosts.

In the midwestern USA, prescribed fire is commonly used to manage oak woodlands and goals often include managing understory vegetation and increasing ground flora diversity [35,36]. Surface fires consume accumulated leaf litter, detritus and a portion of the ground and shrub layer vegetation. Bare ground provides a favorable substrate for seedling regeneration yet a less desirable habitat for ticks [37,38,39]. After burning, vegetation used for questing is reduced and bare ground is exposed, which may reduce habitat quality for ticks. Low intensity burns in woodlands are patchy and may provide refugia for ticks to escape direct mortality caused by fire [40,41].

Prior to this study, records of vector ticks in western Illinois were limited. In addition, little is known about how current management practices (i.e., prescribed fire) affect ticks in oak woodlands. Investigating factors that contribute to tick abundance is critical to understanding spatial patterns of disease risk in the midwestern USA. Our objectives were to (1) examine the effect of spring burning and landscape position on tick abundance, (2) identify habitat variables that are likely to affect tick abundance, and (3) determine which weather variables affect tick collection and if these variables are specific to life stage.

## 2. Materials and Methods

### 2.1. Study Site

The study was conducted in an open oak woodland barren complex at Western Illinois University’s Alice L. Kibbe Field Station located in Warsaw (40.3650° N, 91.4075° W), in Hancock County, IL, USA. The field station consists of ~90 ha owned by Western Illinois University and is adjacent to ~520 ha owned by the Illinois Department of Natural Resources. These areas are comprised of multiple habitat types including, oak-hickory woodlands, early successional woodlands, oak barrens, floodplain forests, restored tallgrass prairies and hill prairies. The Illinois Natural Areas Inventory (INAI) considers the study site to be a community high in floristic quality and fauna [42].

Xeric sites were dominated by several overstory hardwood tree species including white oak (*Quercus alba* L.), post oak (*Q. stellata* Wangenh.), mockernut hickory (*Carya tomentosa* Nutt.), and black oak (*Q. velutina* Lam.). Mesic stands were dominated by northern red oak (*Quercus rubra* L.), white ash (*Fraxinus americana* L.), wild black cherry (*Prunus serotina* Ehrh), basswood (*Tilia americana* L.), sugar maple (*Acer saccharrum* Marshall) and American elm (*Ulmus americana* L.). Common understory trees and shrubs included redbud (*Cercis canadensis* L.), hophornbeam (*Ostrya virginiana* (Mill.) K. Koch), roughleaf dogwood (*Cornus drummondii* C.A. Mey.), prickly ash (*Zanthoxylum americanum* Mill.) and Allegheny blackberry (*Rubus allegheniensis* Porter). Dominant understory vegetation included pointed-leaved tick trefoil [*Desmodium glutinosum* (Muhl. Ex Willd.) Alph. Wood.], beggar’s lice (*Hackelia virginiana* L.), white snakeroot (*Ageratina altissima* L.), clustered black snakeroot [*Sanicula odorata* (Raf.) K.M. Pryer and L.R. Phillippe], elmleaf goldenrod, [*Solidago ulmifolia* (Muhl.) ex Willd.], nodding fescue [*Festuca subverticillata* (Pers.) Alexeev], Virginia creeper (*Parthenocissus quinquefolia* L.) and Pennsylvania sedge (*Carex pensylvanica* Lam.).

The entire study site was last burned in 2004 (B04) and additional burns took place in spring of 2014 (B14) and 2015 (B15). Burns were considered low intensity according to Whelan [43] because most flame heights were less than 1 m and plant mortality was limited to the understory vegetative community. Ten 40 m transects were established and georeferenced within each treatment (B04, B14 and B15) and varied among slope position and aspect (Table 1).

### 2.2. Tick Collection

Ticks were collected during two consecutive years (9 May 2015 until 30 October 2015 and 22 April 2016 until 4 November 2016), every two weeks, when vegetation was dry between 1200 and 1800 h. We used a flagging method to collect ticks that was similar to the method used by Rulison [44]. However, instead of using a wooden dowel, we used a bamboo stem that was not held in place with clamps; it was guided through a tightly stitched pocket. At the beginning of each transect, a sealed bag containing a 1 m^2^ flannel cloth was opened and threaded onto a bamboo stem. Flagging began at 0 m along one side of each transect. At 40 m, the same flag was flipped over so the reverse side faced the ground and flagging continued along the opposite side, yielding a total flagged area of 80 m^2^ per transect. When sampling at each transect was complete, the cloth was folded and placed in a labeled, sealable plastic bag. Bags containing flaggings were returned to the lab and frozen at −10 °C for a minimum of 3 days to ensure tick mortality [45]. Flags were cleaned of vegetation and ticks were removed, enumerated, and identified using taxonomic keys [46,47]. DNA was extracted from each tick collected in 2015 and 2016 and is available for testing veracity of species identifications. It is stored, along with all collection data, at the Vector Biology Initiative Laboratory, Department of Biological Sciences, Western Illinois University, IL, USA.

### 2.3. Weather Data

Prior to tick sampling, weather data from the National Weather Service (NWS) were recorded from the nearest weather station located at Keokuk Municipal Airport, Keokuk, IA (KEOK). These data included temperature, wind speed and relative humidity. Cloud cover was observed and noted by researchers at the site during 2016; cloud cover was not recorded in 2015. We recorded and averaged total precipitation for the two weeks prior to tick collection dates and included the day of sampling using data from the National Centers for Environmental Information (NOAA) [48].

### 2.4. Vegetation Sampling

During late August and September of 2015 and 2016, we used the modified Daubenmire cover scale (1 = 0–5%, 2 = 5–25%, 3 = 25–50%, 4 = 50–75%, 5 = 75–95%, and 6 = 95–100%) to assign cover classes to individual ground flora species, and to measure percent bare ground, leaf litter and coarse woody debris [49]. Tallest vegetation height was recorded every 5 m and averaged for each transect. A GRS^TM^ densitometer (Arcarta, CA, USA) was used to view canopy coverage at 16 points along the midline of each transect. During 2016, tree species found within the 4 by 40 m transect with a diameter at breast height (DBH) >5 cm were considered trees and identified to species [50].

### 2.5. Statistical Analyses

Prior to analyses we calculated mean midpoint cover values for vegetation variables by growth form (annual, biennial, perennial, grass, sedge, shrub, and vine) on each transect. We additionally calculated mean garlic mustard [*Alliaria petiolata* (M. Bieb.) Cavara and Grande] cover, Shannon diversity, Pielou’s evenness, total cover, canopy cover, vegetation height, percent leaf litter, bare ground, coarse woody debris, and total basal area as continuous predictor variables. Categorical predictor variables included treatment (B04, B14, and B15), aspect, transect position (high or low slope) and year. We only conducted analyses on *A. americanum* because it was the most frequently collected tick and sample sizes of other species were small. For analyses of habitat variables, we summed ticks collected on each transect per year for adult and nymphal life stages, and total ticks (all life stages). Larval ticks were considered present (1) or absent (0) because larval ticks are often found in large clusters that may skew the data [31]. Weather analyses were conducted on presence/absence on each tick life stage, per transect and sample period. Quantile plots and histograms were used to assess distribution of response variables.

We fit mixed models with a negative binomial distribution using package “glmmADMB” [51] to total, adult, and nymphal ticks. Logistic models were used for larval ticks, with predictor as a fixed effect and transect as a random effect to test how treatment and habitat variables affected tick abundance. For categorical predictor variables we also evaluated a predictor-by-year interaction to assess differences among categorical variables between years. We then conducted analysis of variance (ANOVA) on each model using package “car” [52]. We used package “lsmeans” [53] to conduct post hoc tests of least-square means with Tukey adjustment for categorical treatment variables that were significant. Spearman’s rank correlation was used to determine if there were linear correlations between response and predictor variables. For weather analyses we used function “glm” to conduct logistic regression on total ticks and each life stage, with a binomial logit link. Weather analyses were conducted for 2015 and 2016 separately. We used Wald chi-square analysis to test for differences of response and continuous predictor variables in logistic models. *T*-tests were used to assess continuous weather variables between years. All statistical analyses were conducted in program R ver. 3.4.1 [54], and all graphics were produced using SigmaPlot ver. 10.0 [55].

## 3. Results

### 3.1. Treatment and Landscape

We collected a total of 2788 *A. americanum* during 2015 and 2016. Ticks collected from B04 made up 51% of the collection (*n* = 1433), B14 37% (*n* = 1045), and B15 11% (*n* = 307). Of these ticks, 2% (*n* = 67) were adults, 4% (*n* = 107) were nymphs, and 93% (*n* = 2614) were larvae. We collected 54 *I. scapularis* of which 4% (*n* = 2) were adults, 22% (*n* = 12) were nymphs, and 74% (*n* = 40) were larvae. We also collected 23 *D. variabilis* of which 74% (*n* = 17) were adults, 9% (*n* = 2) were nymphs, and 17% (*n* = 4) were larvae.

**Figure 1:**
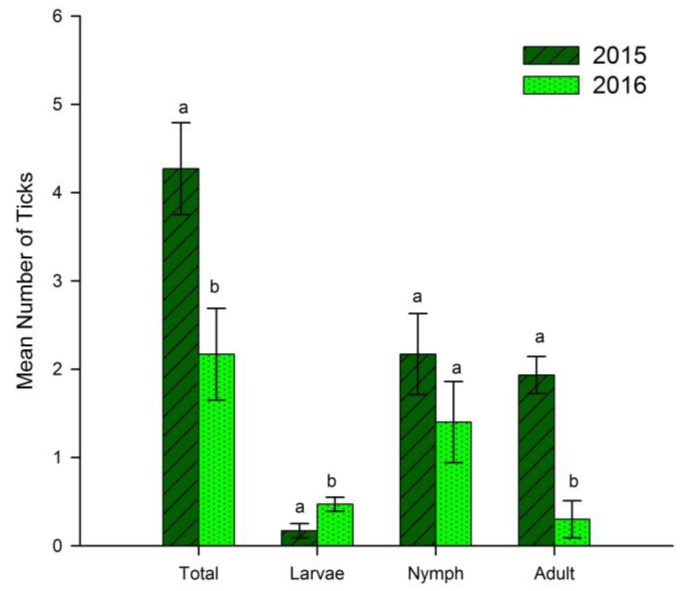
Mean counts of *Amblyomma americanum* (L.) by life stage, collected from transects in 2015 (*n* = 30 transects) and 2016 (*n* = 30 transects) at Alice L. Kibbe Field Station in Hancock County, west-central Illinois. Bars superscripted with different letters indicate significant differences (*p* < 0.05) between years as indicated by one-way ANOVA. Larval ticks were considered present (1) or absent (0), means of presence and absence are depicted. Error bars represent one standard error.

Total ticks, nymphal and larval *A. americanum* did not differ among burn treatment units (*F* = 0.90; *df* = 2, 53; *p* = 0.41: *F* = 1.56; *df* = 2, 53; *p* = 0.22: *F* = 0.05; *df* = 2, 53; *p* = 0.95, respectively). However, adult tick abundance was marginally higher on B14 units (mean = 1.6, SE = 0.4) compared to B04 (mean = 0.90, SE = 0.2) and B15 (mean = 0.85, SE = 0.3) (*F* = 2.948; *df* = 2, 53; *p* = 0.06). Additionally, slope and aspect showed no significant relationship among total ticks, adults, nymphs, or larvae. Total ticks (*F* = 10.158; *df* = 1, 57; *p* = 0.002), larval ticks (*F* = 5.826; *df* = 1, 57; *p* = 0.02), and adult ticks (*F* = 24.448; *df* = 1, 57; *p* ≤ 0.0001) differed between years (Figure 1).

### 3.2. Habitat

Total tick abundance differed with respect to Shannon-diversity index for vegetation, perennial cover, percent bare ground and coarse woody debris (Table 2). Adult tick abundance differed with respect to total vegetation cover and leaf litter cover (Table 2). Nymphal tick abundance differed with respect to annual cover; however, there was no linear trend. Nymphal tick abundance also differed with respect to percent bare ground (Table 2). Although not significant in ANOVA tests, Spearman rank correlation indicated significant negative linear relationships between total ticks and biennial cover, while adult ticks were negatively associated with biennial and annual cover and percent coarse woody debris (Table 2).

**Figure 2:**
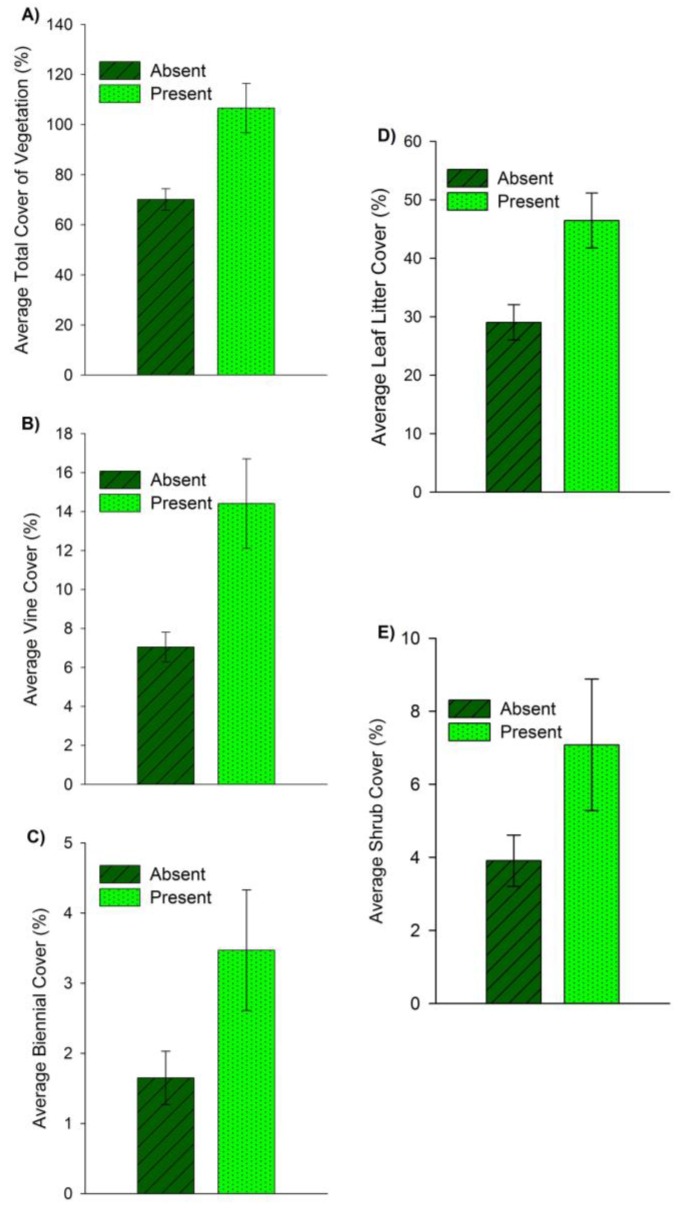
Comparison of larval *Amblyomma americanum* (L.) presence (*n* = 19) and absence (*n* = 41) on transects during all sample periods with respect to (**A**) Average total vegetation cover, (**B**) Average vine cover, (**C**) Average biennial cover, (**D**) Average leaf litter cover, and (**E**) Average shrub cover. Ticks were collected in 2015 and 2016 at Alice L. Kibbe Field Station in Hancock County, west-central Illinois. Presence indicates ≥ 1 larval tick was collected on a transect during the sample periods; absence indicates no larval ticks were collected.

Larval tick presence increased with respect to average total cover (Figure 2A), vine cover (Figure 2B), biennial cover (Figure 2C), leaf litter cover (Figure 2D), and was marginally nonsignificant for shrub cover (Figure 2E; Table 3).

### 3.3. Weather

In 2015, ticks were more likely to be collected when relative humidity was higher (χ^2^ = 8.2, *df* = 1, *p* = 0.004) and with more precipitation occurring two weeks prior to sampling (χ^2^ = 25.2, *df* = 1, *p* < 0.001) but less likely as the sample season progressed (χ^2^ = 59.9, *df* = 1, *p* < 0.001). Adult ticks were more likely to be collected when relative humidity (χ^2^ = 7.2, *df* = 1, *p* = 0.007) and precipitation (χ^2^ = 7.7, *df* = 1, *p* = 0.005) was higher and decreased as the sample season progressed (χ^2^ = 65.0, *df* = 1, *p* < 0.001). Nymphal tick collection increased with temperature (χ^2^ = 3.0, *df* = 1, *p* = 0.008), relative humidity (χ^2^ = 5.3, *df* = 1, *p* = 0.02), and precipitation (χ^2^ = 26.5, *df* = 1, *p* < 0.001) but decreased as the sample season progressed (χ^2^ = 22.4, *df* = 1, *p* < 0.001). Larval ticks were less likely to be collected with increasing precipitation (χ^2^ = 3.9, *df* = 1, *p* = 0.05; Table 4).

In 2016, total tick and nymphal collection increased with temperature (χ^2^ = 7.7, *df* = 1, *p* = 0.006; χ^2^ = 8.9, *df* = 1, *p* = 0.003, respectively). Adult ticks were less likely to be collected with increasing relative humidity (χ^2^ = 6.0, *df* = 1, *p* = 0.01) and as the sample season progressed (χ^2^ = 9.0, *df* = 1, *p* = 0.003). Nymphal collection decreased as the sample season progressed (χ^2^ = 18.2, *df* = 1, *p* < 0.001) but increased with wind (χ^2^ = 4.0, *df* = 1, *p* = 0.046). Nymphal tick collection also differed with respect to cloud cover (χ^2^ = 7.8, *df* = 1, *p* = 0.02) and nymphs were less likely to be collected on clear days or days with full cloud cover. Larval tick collection increased as the sample period progressed (χ^2^ = 21.1, *df* = 1, *p* < 0.001), but larvae were less likely to be collected on windy days (χ^2^ = 7.5, *df* = 1, *p* = 0.006). Larval ticks were more likely to be collected on full cloud cover days (χ^2^ = 7.7, *df* = 1, *p* = 0.005) than clear or partly cloudy days (Table 5). Relative humidity was higher in 2015 (mean = 75.1, SE = 3.8) compared to 2016 (mean = 58.0, SE = 2.7; *T* = 3.7, *df* = 24, *p* = 0.001). All other climate variables did not differ between years.

## 4. Discussion

### 4.1. Treatment and Landscape

Of the three species of ticks collected, *A*. *americanum* made up the majority (97%), which supports previous research documenting their aggressive questing nature and preference for secondary growth woodland habitat [11,14]. Total *A. americanum* and all life stages collected did not differ among burn treatments, which suggests that the low intensity burning conducted at the site had a limited effect on the number of ticks collected. Additionally, it is possible that hosts harboring ticks could have immigrated to recently burned areas in search of mast and young vegetation for food, and consequently, ticks could have dropped off in these areas and re-established. Previous research describing the effects of fire on ticks are conflicting; some studies show an initial decrease in ticks with populations rebounding in subsequent years [27,31,56,57,58,59]. Our study and several others have suggested that tick presence may not be significantly affected by burning [39,40,60]. In the future, seasonality of burns should be investigated to compare the effects of warm season (fall) and cool season (spring) burns on tick presence.

Interestingly, when total ticks, adults, nymphs, and larvae were compared in relation to landscape position (slope and aspect) ticks were not distributed differently, which implies that hosts had no preference regarding slope or aspect. Lack of effect with respect to slope could have been caused by low variation between transects due to spatial proximity and minimal elevation differences at the site. Steep slopes are probably less attractive to hosts and consequently may have fewer ticks [61]. We also expected some variation in tick presence among aspects, yet none were found. South-facing aspects are warmer and drier, whereas north facing aspects are cooler with more moisture. Variations in moisture and temperature also contribute to differences in vegetation communities.

When each life stage was compared between sample years, nymphs collected were similar between years, but all other life stages were significantly different between years (Figure 1). A model developed by Haile and Mount [62] for simulating environmental variables has been used to investigate why ticks vary among years. Using this model, it was determined that annual variation of ticks could be due to host-finding rate (based on an interaction of tick life stage, day length, and host density) and weather (temperature and relative humidity) [27]. With regards to our study, we examined how tick life stages varied between two years and found that life stages varied seasonally and annually. Similarly, we found that the effect of weather variables was life stage-specific and affected tick collection. Nymphs collected may have been similar in both years simply by chance.

### 4.2. Habitat

In a previous study, plant diversity was found to be an insignificant predictor of *A. americanum* adult and nymphal abundance [63]. Our results indicate that plant diversity (based on Shannon diversity index) had a weak negative correlation to total *A. americanum* abundance. Unmanaged areas typically have more invasive vegetation that decreases diversity, provides food and cover for hosts, and may provide a favorable microhabitat with a more constant relative humidity [20,64]. Habitats with high abundances of exotic, invasive species such as bush honeysuckle [*Lonicera macckii* (Rupr.) Herder], Japanese barberry [*Berberis thunbergii* de Candolle] and multiflora rose [*Rosa multiflora* Thunb.] have been shown to support more ticks [20,65,66]. Additionally, total ticks showed a weak negative correlation to perennial cover, which likely provided questing habitat and contributed to the overall diversity at the site (Table 2). Thus, it would be ecologically plausible if ticks were negatively associated with areas with high diversity.

Total *A. americanum* and nymphs collected were negatively associated with percent bare ground. These results suggest that more bare ground reduces tick abundance and creates an unfavorable environment for ticks. Conversely, adult abundance differed in respect to leaf litter cover with tick collection decreasing slightly as litter cover increased. Schulze [67] found that nymphal *A. americanum* were more tolerant than *I. scapularis* to dry conditions. Additionally, since adults have a smaller surface area to volume ratio than larvae, they may be able to tolerate less leaf litter [19,68]. Other studies that have examined litter cover, grass cover and litter mass have showed positive associations with tick abundance, which suggests that ground cover is important to the life cycle of *A. americanum* [31,39]. Total cover differed in respect to adult *A. americanum*; however, there was no significant linear relationship.

Larval *A. americanum* presence was associated with several vegetation variables which included: biennial cover, vine cover, shrub cover, leaf litter cover and total cover. As these vegetation variables increased, larval tick presence increased, suggesting that larval ticks may be affected by differences in vegetation to a greater degree than other life stages, perhaps due to their surface area to volume ratio, or that hosts prefer using areas with greater vegetation cover. Biennial plants invest their energy in basal leaves during the first year of growth and provide ground cover that could protect ticks from desiccation [69]. Leaf litter cover and total vegetation cover may be even more important to larval ticks due to their strong moisture requirements [33]. Shrub cover was marginally insignificant but likely provided cover for *O. virginianus* and shade that contributes to a mesic environment [20,70]. Vines such as Virginia creeper [*Parthenocissus quinquefolia* (L.) Planch], *Vitis* spp., poison ivy [*Toxicodendron radicans* (L.) Kuntze], common periwinkle, *Vinca minor* (L.), and *Smilax* spp. are ubiquitous at the study site and have been found in gut diet analyses of white-tailed deer [70]. Documenting species browsed and camera-trap surveys along transects should be included in future studies to further examine the relationship between host dynamics and tick presence.

Previously, tick sampling biases have been found between species and life stages [67,71]. Presence of coarse woody debris had a weak negative association with total ticks collected, which could have been due to the sampling method used. We used a flagging method to collect ticks and previous studies have shown that CO_2_ traps collect more *A. americanum* [71]. It is possible that flagging over debris could be less efficient and result in the loss of ticks.

### 4.3. Weather

*A. americanum* have distinct seasonal trends in the midwestern USA. Adult ticks in the Midwest are most active from May through July, nymphs from May through August, and larvae July through September [22]. Similar seasonal tick activity patterns were observed during our study. As the sample period progressed in 2015, total, adult and nymphal collection decreased. In 2016, nymphal collection only decreased and conversely, larval tick collection increased as the sampling period progressed. Mean monthly temperatures were higher in 2016 during months larval ticks were collected, which would have caused soil temperatures to stay warmer. Increases in soil temperature have been shown to influence the timing of larval *A. americanum* hatch [72].

Studies have shown that *A. americanum* prefer a relative humidity of 85% or greater for reproduction and molting success [73,74]. *A. americanum* are more dependent on humidity than *D. variabilis* [75]. We expected ticks to quest when humidity was high because moisture from the atmosphere would be readily available. As relative humidity increased during 2015, more total, adult and nymphal ticks were collected; conversely during 2016, fewer adult and larval ticks were collected as relative humidity increased. These results were inconsistent between years and could have been influenced by temporal differences in precipitation. Precipitation and moisture have previously been shown to highly influence tick activity [61,76]. All adult and nymphal ticks were collected in May, June and early July of both years and total precipitation during these months in 2015 was over double that during these same months in 2016 (50.75 cm and 18.75 cm, respectively), based on data from the National Centers for Environmental Information (NOAA) [48]. Collections during 2015 yielded more adult and nymphs than in 2016 and higher precipitation was a likely contributor. In future studies, it would be beneficial to have weather data collected at each transect, due to the high sensitivity of ticks to microclimate.

Other weather variables such as wind and cloud cover were significant for nymphal and larval stages only during 2016—however, confidence intervals overlapped zero and trends were not consistent between years, making them less reliable predictors of tick collection. Of these other significant variables, wind appeared to be the only reliable predictor for larval tick collection. Results showed that an increase in wind decreased the likelihood of collecting larval ticks, which is not surprising considering their small size and tendency to lose moisture quickly. Larval ticks are more likely to be negatively affected by increased wind speed due to their greater susceptibility of desiccation [32].

## 5. Conclusions

With tick-borne diseases on the rise in Illinois and worldwide, our results are important because we were able to document additional records for vector ticks in Illinois. These data serve as a baseline for the effects of low intensity burns, habitat, and weather patterns on tick presence in a midwestern USA oak woodland community. In areas where vector tick collection is higher, disease risk for those who are active in outdoor environments is likely greater. Although significant habitat variables were found, most of these were weakly correlated to *A. americanum* presence or abundance and several were inconsistent between years. Conversely, the odds of encountering larvae with respect to significant habitat variables were stronger. Thus, it is vital to continue long-term monitoring of factors that may contribute to tick presence and abundance which include both spatial and temporal trends, particularly in light of future effects of climate change on these factors.

## Figures and Tables

**Table 1:** Transects located at the Alice L. Kibbe Field Station, Hancock Co., IL, in west-central Illinois showing treatment, aspect, and transect position. Transects considered “High” were up slope, whereas those considered “Low” were downslope.

Transect	Treatment	Aspect	Position
Bluff T1	B15	South	High
Bluff T2	B15	South	Low
CC1 T1	B15	West	High
CC1 T2	B15	West	High
CC2 T1	B15	West	High
CC2 T2	B15	West	High
NWBT1	B04	Northeast	High
NWB T2	B04	Northeast	Low
NWBPT1 T1	B04	Northeast	High
NWBPT1 T2	B04	Northeast	Low
NWBPT2 T1	B14	Northeast	High
NWBPT2 T2	B14	Northeast	Low
NWBPT3 T1	B15	Northeast	High
NWBPT3 T2	B15	Northeast	Low
PGIW T1	B14	Southwest	High
PGIW T2	B14	Southwest	Low
PGPO T1	B04	Southwest	High
PGPO T2	B04	Southwest	Low
PGPT T1	B14	West	High
PGPT T2	B14	West	Low
PGWO T1	B04	Southwest	High
PGWO T2	B04	Southwest	Low
PNDG T1	B15	Southwest	High
PNDG T2	B15	Southwest	Low
PNDPT T1	B04	Southwest	High
PNDPT T2	B04	Southwest	Low
UPOS T1	B14	Southwest	High
UPOS T2	B14	Southwest	Low
WNCK T1	B14	Southwest	High
WNCK T2	B14	Southwest	High

**Table 2:** Results from ANOVA and Spearman rank correlation of *Amblyomma americanum* (L.) by life stage and all habitat variables.

		Total			Adult			Nymph		
Variable	DF	*F*	*p*-Value	Spearman ρ	*F*	*p*-Value	Spearman ρ	*F*	*p*-Value	Spearman ρ
Shannon-diversity	1,56	3.9	0.05	**−0.30 ^a^**	2.1	0.15	−0.18	1.4	0.24	−0.15
Evenness	1,56	2.1	0.16	−0.15	0.44	0.51	−0.08	2.2	0.15	−0.10
Total Cover	1,56	2.1	0.15	−0.18	4.3	0.04	−0.20	0.49	0.49	−0.08
Perennial Cover	1,56	5.3	0.03	**−0.26**	3.7	0.06	−0.22	3.3	0.08	−0.19
Biennial Cover	1,56	0.64	0.43	**−0.31**	2.6	0.11	**−0.38**	<0.01	0.98	−0.12
Annual Cover	1,56	0.97	0.33	−0.20	0.46	0.50	**−0.27**	5.0	0.03	0.0
Grass Cover	1,56	0.27	0.61	0.12	0.17	0.68	0.03	0.99	0.32	0.21
Sedge Cover	1,56	0.01	0.93	−0.01	1.34	0.25	0.02	0.34	0.56	−0.02
Shrub Cover	1,56	0.14	0.71	0.12	0.55	0.46	0.06	1.30	0.26	0.13
Vine Cover	1,56	0.64	0.43	−0.14	2.46	0.12	−0.18	0.82	0.37	−0.14
Garlic Mustard Cover	1,56	0.03	0.86	−0.20	0.74	0.39	−0.20	0.19	0.67	−0.11
Mean Vegetation Height	1,56	0.31	0.58	0.15	0.86	0.36	0.14	<0.01	0.99	0.10
Canopy Cover	1,56	1.36	0.25	−0.19	0.89	0.35	−0.11	1.29	0.26	−0.16
Leaf Litter Cover	1,56	0.03	0.86	−0.01	4.68	0.03	**−0.26**	0.26	0.62	0.10
Course Woody Debris Cover	1,56	6.89	0.01	**−0.40**	0.70	0.41	**−0.49**	1.04	0.31	−0.14
Bare Ground %	1,56	7.58	0.008	**−0.36**	2.06	0.16	−0.13	3.87	0.05	**−0.26**
Basal Area	1,56	0.03	0.86	0.0	0.24	0.62	0.13	<0.01	0.95	−0.04

**^a^** Bold values indicate significant association (*p* < 0.05) as indicated by Spearman rank correlation.

**Table 3:** Coefficient (β), standard error (SE), 95% confidence limits, and chi-square test of significant habitat variables on presence of larval *Amblyomma americanum* (L.) collected in 2015 and 2016.

Parameter	*n*	β	SE	95% Confidence Limits		χ^2^	*p*-Value
				LCL	UCL		
Total Cover	60	0.02	0.008	0.002	0.03	4.7	0.03
Biennial	60	0.19	0.10	0.006	0.38	4.1	0.04
Vine	60	0.17	0.06	0.05	0.28	8.2	0.004
Shrub	60	0.09	0.05	−0.009	0.19	3.2	0.07
Leaf Litter	60	0.04	0.01	0.013	0.07	7.9	0.005

**Table 4:** Coefficient (β), standard error (SE) and 95% confidence limits of significant weather variables on presence of *Amblyomma americanum* (L.) collected in 2015.

Response	Parameter	Level (a)	*n*	β	SE	95% Confidence Limits	
						LCL	UCL
Total	Relative Humidity	75.1	420	0.0263	0.01	0.01	0.05
	Sample Period	7.5	420	−0.2609	0.04	−0.34	−0.19
	Precipitation	1.7	420	0.3843	0.08	0.23	0.54
Adult	Relative Humidity	75.1	420	0.0337	0.01	0.01	0.06
	Sample Period	7.5	420	−0.4302	0.07	−0.58	−0.30
	Precipitation	1.7	420	0.2764	0.10	0.08	0.47
Nymphs	Temperature	19.4	420	0.0448	0.03	−0.01	0.10
	Relative Humidity	75.1	420	0.0288	0.01	0.004	0.06
	Sample Period	7.5	420	−0.2083	0.05	−0.31	−0.12
	Precipitation	1.7	420	0.5165	0.10	0.32	0.72
Larvae	Precipitation	1.7	420	−0.5690	0.34	−1.4	−0.004

(**a**) Mean values were calculated for continuous variables.

**Table 5:** Coefficient (β), standard error (SE) and 95% confidence limits of significant weather variables on presence of *Amblyomma americanum* (L.) collected in 2016.

Response	Parameter	Level (a)	*n*	β	SE	95% Confidence Limits	
						LCL	UCL
Total	Temperature	22.2	450	0.0773	0.03	0.0	0.14
	Precipitation	1.4	450	0.3962	0.17	0.05	0.74
Adult	Relative Humidity	58.0	450	−0.1049	0.05	−0.21	−0.02
Nymphs	Temperature	22.2	450	0.1033	0.04	0.03	0.18
	Sample Period	8	450	−0.1894	0.05	−0.29	−0.10
	Wind	6.9	450	0.1211	0.06	0.00	0.25
	Cloud Cover	Clear	180	−2.26	0.25	−2.80	−1.79
		Partly	120	0.063	0.37	−0.68	0.80
		Full	150	−1.403	0.64	−2.87	−0.28
Larvae	Sample Period	8	450	0.24	0.06	0.13	0.36
	Wind	6.9	450	−0.1653	0.06	−0.29	−0.05
	Precipitation	1.4	450	0.6142	0.26	−0.28	0.63
	Cloud Cover	Clear	180	−2.732	0.31	−3.41	−2.17
		Partly	120	−0.8653	0.59	−0.22	1.48
		Full	150	0.6241	0.43	−2.17	0.23

(**a**) Mean values were calculated for continuous variables.

## References

[1] Jongejan F., Uilenberg G. (2004). The global importance of ticks. Parasitology.

[2] Stromdahl E.Y., Hickling G.J. (2012). Beyond Lyme: Aetiology of tick-borne human diseases with emphasis on the south-eastern United States. Zoonoses Public Health.

[3] De la Fuente J., Estrada-Pena A., Venzal J.M., Kocan K.M., Sonenshine D.E. (2008). Overview: Ticks as vectors of pathogens that cause disease in humans and animals. Front. Biosci..

[4] Dantas-Torres F., Chomel B.B., Otranto D. (2012). Ticks and tick-borne diseases: A One Health perspective. Trends Parasitol..

[5] Tulloch J.S.P., Mcginley L., Sanchez-Vizcaino F., Medlock J.M., Radford A.D. (2017). The passive surveillance of ticks using companion animal electronic health records. Epidemiol. Infect..

[6] Kilpatrick A.M., Dobson A.D., Levi T., Salkeld D.J., Swei A., Ginsberg H.S., Kjemtrup A., Padgett K.A., Jensen P.M., Fish D. (2017). Lyme disease ecology in a changing world: Consensus, uncertainty and critical gaps for improving control. Philos. Trans. R. Soc. B.

[7] Luckhart S., Mullen G.R., Wright J.C. (1991). Etiologic agent of Lyme disease, *Borrelia burgdorferi*, detected in ticks (Acari: Ixodidae) collected at a focus in Alabama. J. Med. Entomol..

[8] Gilliam M.E. (2017). The Influence of Prescribed Fire on Tick Abundance and *Borrelia burgdorferi* Prevalence in Amblyomma americanum. Master’s Thesis.

[9] Schulze T.L., Bowen G.S., Bosler E.M., Lakat M.F., Parkin W.E., Altman R., Ormiston B.G., Shisler J.K. (1984). *Amblyomma americanum*: A potential vector of Lyme disease in New Jersey. Science.

[10] Burkhalter K.L., Harmon J.R., Ashley D.C., Panella N.A., Lambert A., Savage H.M., Nicholson W.L., Lash R.R., Godsey M.S. (2013). First detection of Heartland virus (Bunyaviridae: Phlebovirus) from field collected arthropods. Am. J. Trop. Med. Hyg..

[11] Childs J.E., Paddock C.D. (2003). The ascendancy of *Amblyomma americanum* as a vector of pathogens affecting humans in the United States. Annu. Rev. Entomol..

[12] Masters E.J., Grigery C.N., Masters R.W. (2008). STARI, or Masters Disease: Lone star tick–vectored Lyme-like illness. Infect. Dis. Clin. N. Am..

[13] Herrmann J.A., Dahm N.M., Ruiz M.O., Brown W.M. (2014). Temporal and spatial distribution of tick-borne disease cases among humans and canines in Illinois (2000-2009). Environ. Health Insights.

[14] Goddard J., Varela-Stokes A.S. (2009). Role of the lone star tick, *Amblyomma americanum* (L.), in human and animal diseases. Vet. Parasitol..

[15] Monzón J.D., Atkinson E.G., Henn B.M., Benach J.L. (2016). Population and evolutionary genomics of *Amblyomma americanum*, an expanding arthropod disease vector. Genome Biol. Evol..

[16] Stegall-Faulk T., Clark D.C., Wright S.M. (2003). Detection of *Borrelia lonestari* in *Amblyomma americanum* (Acari: Ixodidae) from Tennessee. J. Med. Entomol..

[17] Springer Y.P., Eisen L., Beati L., James A.M., Eisen R.J. (2014). Spatial distribution of counties in the continental United States with records of occurrence of *Amblyomma americanum* (Ixodida: Ixodidae). J. Med. Entomol..

[18] Ogden N.H., Trudel L., Artsob H., Barker I.K., Beauchamp G., Charron D.F., Drebot M.A., Galloway T.D., O’Handley R., Thompson R.A. (2006). *Ixodes scapularis* ticks collected by passive surveillance in Canada: Analysis of geographic distribution and infection with Lyme borreliosis agent *Borrelia burgdorferi*. J. Med. Entomol..

[19] Stein K.J., Waterman M., Waldon J.L. (2008). The effects of vegetation density and habitat disturbance on the spatial distribution of ixodid ticks (Acari: Ixodidae). Geospat. Health.

[20] Allan B.F., Goessling L.S., Storch G.A., Thach R.E. (2010). Blood meal analysis to identify reservoir hosts for *Amblyomma americanum* ticks. Emerg. Infect. Dis..

[21] Clymer B.C., Howell D.E., Hair J.A. (1970). Animal hosts of economically important ticks (Acarina) in east-central Oklahoma. Ann. Entomol. Soc. Am..

[22] Kollars T.M., Oliver J.H., Durden L.A., Kollars P.G. (2000). Host association and seasonal activity of *Amblyomma americanum* (Acari: Ixodidae) in Missouri. J. Parasitol..

[23] Paddock C.D., Yabsley M.J. (2007). Ecological havoc, the rise of white-tailed deer, and the emergence of *Amblyomma americanum*-associated zoonoses in the United States. Curr. Top. Microbiol. Immunol..

[24] Gill R.M.A., Beardall V. (2001). The impact of deer on woodlands: The effects of browsing and seed dispersal on vegetation structure and composition. Forestry.

[25] Abbas F., Morellet N., Hewison A.J.M., Merlet J., Cargnelutti B., Lourtet B., Angibault J.-M., Daufresne T., Aulagnier S., Verheyden H. (2011). Landscape fragmentation generates spatial variation of diet composition and quality in a generalist herbivore. Oecologia.

[26] Nixon C.M., Hansen L.P., Brewer P.A., Chelsvig J.E. (1991). Ecology of white-tailed deer in an intensively farmed region of Illinois. Wildl. Monogr..

[27] Davidson W.R., Siefken D.A., Creekmore L.H. (1994). Seasonal and annual abundance of *Amblyomma americanum* (Acari: Ixodidae) in central Georgia. J. Med. Entomol..

[28] Nowacki G.J., Abrams M.D. (2008). The demise of fire and “mesophication” of forests in the eastern United States. Bioscience.

[29] Procházka J., Brom J., Št’astný J., Pecharová E. (2011). The impact of vegetation cover on temperature and humidity properties in the reclaimed area of a brown coal dump. Int. J. Min. Reclam. Environ..

[30] Estrada-Peña A., de la Fuente J. (2014). The ecology of ticks and epidemiology of tick-borne viral diseases. Antivir. Res..

[31] Gleim E.R., Conner L.M., Berghaus R.D., Levin M.L., Zemtsova G.E., Yabsley M.J. (2014). The phenology of ticks and the effects of long-term prescribed burning on tick population dynamics in Southwestern Georgia and Northwestern Florida. PLoS ONE.

[32] Robertson A.S., Patrick C.D., Semtner P.J., Hair J.A. (1975). The ecology and behavior of the lone star tick (Acarina: Ixodidae). VI. Response of unfed adults to certain environmental parameters. J. Med. Entomol..

[33] Gray J.S., Dautel H., Estrada-Peña A., Kahl O., Lindgren E. (2009). Effects of climate change on ticks and tick-borne diseases in Europe. Interdiscip. Perspect. Infect. Dis..

[34] Schulze T.L., Jordan R.A., Hung R.W. (2002). Effects of microscale habitat physiognomy on the focal distribution of *Ixodes scapularis* and *Amblyomma americanum* (Acari: Ixodidae) nymphs. Environ. Entomol..

[35] Peterson D.W., Reich P.B. (2001). Prescribed fire in oak savanna: Fire frequency effects on stand structure and dynamics. Ecol. Appl..

[36] Scharenbroch B.C., Nix B., Jacobs K.A., Bowles M.L. (2012). Two decades of low-severity prescribed fire increases soil nutrient availability in a Midwestern, USA oak (*Quercus(*) forest. Geoderma.

[37] Barnes T.A., Van Lear D.H. (1998). Prescribed fire effects on advanced regeneration in mixed hardwood stands. South. J. Appl. For..

[38] Scasta J.D. (2015). Fire and parasites: An under-recognized form of anthropogenic land use change and mechanism of disease exposure. Ecohealth.

[39] Willis D., Carter R., Murdock C., Blair B. (2012). Relationship between habitat type, fire frequency, and *Amblyomma americanum* populations in east-central Alabama. J. Vector Ecol..

[40] Jacobs K.A., Nix B., Scharenbroch B.C. (2015). The effects of prescribed burning on soil and litter invertebrate diversity and abundance in an Illinois oak woodland. Nat. Areas J..

[41] Jenkins S.E. (1997). Spatial Demography of an Ozark Savanna. Ph.D. Thesis.

[42] Illinois Natural Areas Inventory. https://www.dnr.illinois.gov/conservation/NaturalHeritage/Documents/Database/INAICountyList.pdf.

[43] Whelan R.J. (1995). The Ecology of Fire.

[44] Rulison E.L., Kuczaj I., Pang G., Hickling G.J., Tsao J.I., Ginsberg H.S. (2013). Flagging versus dragging as sampling methods for nymphal *Ixodes scapularis* (Acari: Ixodidae). J. Vector Ecol..

[45] Burks C.S., Stewart R.L., Needham G.R., Lee R.E. (1996). The role of direct chilling injury and inoculative freezing in cold tolerance of *Amblyomma americanum*, *Dermacentor variabilis* and *Ixodes scapularis*. Physiol. Entomol..

[46] Keirans J.E., Litwak T.R. (1989). Pictorial key to the adults of hard ticks, family Ixodidae (Ixodida: Ixodoidea), east of the Mississippi River. J. Med. Entomol..

[47] Keirans J.E., Durden L.A. (1998). Illustrated key to nymphs of the tick genus *Amblyomma* (Acari: Ixodidae) found in the United States. J. Med. Entomol..

[48] National Centers for Environmental Information [US]. https://www.ncdc.noaa.gov/.

[49] Coulloudon B., Eshelman K., Gianola J., Habich N., Hughes L., Johnson C., Pellant M., Podborny P., Rasmussen A., Robles B. (1999). Sampling Vegetation Attributes.

[50] Glossary of Forestry Terms from the West Virginia Forestry Association. http://www.wvfa.org/pdf/sfi/Glossaryofforestryterms.pdf.

[51] Bolker B., Skaug H., Magnusson A., Nielsen A. (2012). Getting Started with the glmmADMB Package. glmmadmb.r-forge.r-project.org/glmmADMB.pdf.

[52] Fox J., Weisberg S. (2011). An R Companion to Applied Regression.

[53] Lenth R.V. (2016). Least-Squares Means: The *R* Package lsmeans. J. Stat. Softw..

[54] R Development Core Team (2016). R: A Language and Environment for Statistical Computing.

[55] (2007). SigmaPlot.

[56] Allan B.F. (2009). Influence of prescribed burns on the abundance of *Amblyomma americanum* (Acari: Ixodidae) in the Missouri Ozarks. J. Med. Entomol..

[57] Mather T.N., Duffy D.C., Campbell S.R. (1993). An unexpected result from burning vegetation to reduce Lyme disease transmission risks. J. Med. Entomol..

[58] Stafford K.C., Ward J.S., Magnarelli L.A. (1998). Impact of controlled burns on the abundance of *Ixodes scapularis* (Acari: Ixodidae). J. Med. Entomol..

[59] Wilson M.L. (1986). Reduced abundance of adult *Ixodes dammini* (Acari: Ixodidae) following destruction of vegetation. J. Econ. Entomol..

[60] Padgett K.A., Casher L.E., Stephens S.L., Lane A.R.S. (2009). Effect of prescribed fire for tick control in California chaparral. J. Med. Entomol..

[61] Del Fabbro S., Gollino S., Zuliani M., Nazzi F. (2015). Investigating the relationship between environmental factors and tick abundance in a small, highly heterogeneous region. J. Vector Ecol..

[62] Mount G.A., Haile D.G. (1989). Computer simulation of population dynamics of the American dog tick (Acari: Ixodidae). J. Med. Entomol..

[63] Trout Fryxell R.T., Moore J.E., Collins M.D., Kwon Y., Jean-Philippe S.R., Schaeffer S.M., Odoi A., Kennedy M., Houston A.E. (2015). Habitat and vegetation variables are not enough when predicting tick populations in the southeastern United States. PLoS ONE.

[64] Williams S.C., Ward J.S. (2010). Effects of Japanese barberry (Ranunculales: Berberidaceae) removal and resulting microclimatic changes on *Ixodes scapularis* (Acari: Ixodidae) abundances in Connecticut, USA. Environ. Entomol..

[65] Adalsteinsson S.A., D’Amico V., Shriver W.G., Brisson D., Buler J.J. (2016). Scale-dependent effects of nonnative plant invasion on host-seeking tick abundance. Ecosphere.

[66] Williams S.C., Linske M.A., Ward J.S. (2017). Long-term effects of *Berberis thunbergii* (Ranunculales: Berberidaceae) management on *Ixodes scapularis* (Acari: Ixodidae) abundance and *Borrelia burgdorferi* (Spirochaetales: Spirochaetaceae) prevalence in Connecticut, USA. Environ. Entomol..

[67] Schulze T.L., Jordan R.A., Hung R.W. (1997). Biases associated with several sampling methods used to estimate abundance of *Ixodes scapularis* and *Amblyomma americanum* (Acari: Ixodidae). J. Med. Entomol..

[68] Semtner P.J., Barker R.W., Hair J.A. (1971). The ecology and behavior of the Lone Star Tick (Acarina: Ixodidae) II. Activity and survival in different ecological habitats. J. Med. Entomol..

[69] Meekins J.F., McCarthy B.C. (2000). Responses of the biennial forest herb *Alliaria petiolata* to variation in population density, nutrient addition and light availability. J. Ecol..

[70] Sotala D.J., Kirkpatrick C.M. (1973). Foods of white-tailed deer, *Odocoileus virginianus*, in Martin County, Indiana. Am. Midl. Nat..

[71] Solberg V.B., Neidhardt K., Sardelis M.R., Hildebrandt C., Hoffmann F.J., Boobar L.R. (1992). Quantitative evaluation of sampling methods for *Ixodes dammini* and *Amblyomma americanum* (Acari: Ixodidae). J. Med. Entomol..

[72] Patrick C.D., Hair J.A. (1979). Oviposition behavior and larval longevity of the lone star tick, *Amblyomma americanum* (Acarina: Ixodidae), in different habitats. Ann. Entomol. Soc. Am..

[73] Lancaster J.L., McMillan H.L. (1955). The effects of relative humidity on the lone star tick. J. Econ. Entomol..

[74] Adler G.H., Telford S.R., Wilson M.L., Spielman A. (1992). Vegetation structure influences the burden of immature *Ixodes dammini* on its main host, *Peromyscus leucopus*. Parasitology.

[75] Hair J.A., Sauer J.R., Durham K.A. (1975). Water balance and humidity preference in three species of ticks. J. Med. Entomol..

[76] Randolph S.E., Storey K. (1999). Impact of microclimate on immature tick-rodent host interactions (Acari: Ixodidae): Implications for parasite transmission. J. Med. Entomol..

